# Phase-Inversion In Situ Implants for Dental Drug Delivery: A QbD-Guided In Vitro Technological Evaluation

**DOI:** 10.3390/polym18121420

**Published:** 2026-06-07

**Authors:** Elena O. Bakhrushina, Polina S. Sakharova, Mariya V. Kotilevskaya, Iosif B. Mikhel, Galina E. Brkich, Natalya V. Pyatigorskaya, Anzhela S. Brago, Grigory Yu. Evzikov, Yuriy L. Vasiliev

**Affiliations:** 1 Department of Pharmaceutical Technology, A.P. Nelyubin Institute of Pharmacy, I.M. Sechenov First Moscow State Medical University (Sechenov University), Moscow 119048, Russia; bakhrushina_e_o@staff.sechenov.ru (E.O.B.); p.s.sakharova@gmail.com (P.S.S.); m.kotilev@mail.ru (M.V.K.); 2 Department of Industrial Pharmacy, I.M. Sechenov First Moscow State Medical University (Sechenov University), Moscow 119048, Russia; brkich_g_e@staff.sechenov.ru (G.E.B.); pyatigorskaya_n_v@staff.sechenov.ru (N.V.P.); 3 Department of Propaedeutics of Dental Diseases, Institute of Medicine, Peoples’ Friendship University of Russia Named After Patrice Lumumba, Moscow 117198, Russia; anzhela_bogdan@mail.ru; 4 Department of Nervous Diseases and Neurosurgery, N.V. Sklifosovsky Institute of Clinical Medicine, I.M. Sechenov First Moscow State Medical University (Sechenov University), Moscow 119048, Russia; mmaevzikov@mail.ru; 5 Department of Operative Surgery and Topographic Anatomy, N.V. Sklifosovsky Institute of Clinical Medicine, I.M. Sechenov First Moscow State Medical University (Sechenov University), Moscow 119048, Russia; vasilev_yu_l@staff.sechenov.ru

**Keywords:** phase-inversion in situ implant, shellac, quality by design, periodontal drug delivery, solvent screening, critical quality attributes

## Abstract

Phase-inversion in situ implants (PIISIs) represent a versatile polymer platform in which the rational choice of matrix former and solvent system directly governs the macroscopic properties of the resulting depot. This study applied a Quality by Design (QbD) approach to rationalize a bleached shellac–based PIISI, with particular focus on the physicochemical interactions between the polymer and the injection vehicle. Bleached shellac—a natural, low-cost, biodegradable oligomeric resin bearing –COOH, –OH, and ester functional groups—was selected as the matrix former and screened in seven neat solvents and five 1:1 binary combinations at 25% (m/m). Twelve formulations were evaluated against a predefined set of critical quality attributes, including injectability, phase-inversion kinetics, solvent diffusion volume, and implant structure (n = 5 per formulation; mean ± standard deviation (SD); one-way analysis of variance (ANOVA) with Tukey’s post hoc test, *p* < 0.05). Three lead solvent systems—propylene glycol/N-methylpyrrolidone (PG+NMP), PG/dimethyl sulfoxide (PG+DMSO), and DMSO/benzyl alcohol (DMSO+BA)—were identified as those providing an optimal balance between hydrogen-bond donor/acceptor solvation and controlled solvent extraction. In the second stage, shellac concentration (20–35%) was optimized, with 30% shellac in PG+NMP yielding the fastest phase inversion (~50 s), a structurally uniform matrix, and the lowest swelling (22%). A working mechanistic framework consistent with all observed critical quality attribute (CQA) trends in which solvent hydrogen-bond donor/acceptor balance and water miscibility govern implant architecture is proposed, and it is intended as a hypothesis-generating basis for the rational design of PIISI formulations; direct validation by spectroscopic, thermal-analytical, and biological methods is identified as the next step. The developed formulations are presented as a preliminary physicochemical platform; biological validation (in vitro cytocompatibility and inflammatory response assessment) is required before the system can be considered a validated formulation for dental drug delivery.

## 1. Introduction

Phase-inversion in situ forming systems generate a semi-solid or solid implant at the site of administration through diffusion of the organic solvent into the surrounding aqueous tissue fluids, producing a polymer depot that enables sustained release of the active pharmaceutical ingredient (API) [1]. Initiation of the phase transition by solvent exchange is the simplest of all known in situ drug delivery technologies because it relies solely on passive solvent diffusion and is therefore less sensitive to external stimuli than thermo-, pH- or ion-sensitive in situ systems [2]. A phase-inversion in situ implant (PIISI) consists of a biocompatible inert solvent and a polymer matrix former. The polymer must be non-toxic, biodegradable, highly soluble in the chosen solvent, and insoluble in biological fluids in order to precipitate as a stable depot upon contact with tissue.

Phase-inversion systems have been applied in oncology and in the treatment of musculoskeletal disorders, but their most frequent indication is in dental practice [2,3,4,5].

The administration of liquid polymer-based materials that subsequently change their physical state at the site of application is a well-established procedure in dental practice. In recent years, phase-inversion implant technology has expanded rapidly as a drug delivery platform, owing both to the broad range of polymer compositions that can be used and to the more flexible regulatory requirements that apply to local dental administration compared with the stringent standards of parenteral delivery.

In dental applications, PIISI are used most often for drug delivery into the periodontal pocket and less frequently into the alveolar socket. Such compositions have been investigated as carriers for growth factors [6], antibiotics [7,8], anti-inflammatory drugs [9] and analgesics [10]. Continuing interest in the technology is also driven by the long-standing clinical use, for more than a quarter of a century, of a phase-inversion in situ product, Atridox^®^ (TOLMAR Therapeutics Inc., Fort Collins, CO, USA) [11]. Atridox^®^ is built on the Atrigel^®^ delivery platform, a biodegradable liquid matrix consisting of 36.7% (*w*/*w*) poly(DL-lactide) (PLA) dissolved in 63.3% (*w*/*w*) N-methyl-2-pyrrolidone (NMP).

The matrix-forming polymers most frequently considered for PIISI are poly(lactic acid), polylactide, polycaprolactone, N-lauroyl-l-alanine methyl ester, sucrose acetate isobutyrate, borneol, shellac, and PLGA (poly(lactic-co-glycolic acid)), the latter accounting for more than 60% of published studies. The most commonly investigated solvents include NMP, triacetin, triethyl citrate, dimethyl sulfoxide, benzyl benzoate, benzyl alcohol, glycofurol and ethyl lactate [2]. New polymers continue to be evaluated in an effort to develop in situ systems with improved performance [12].

Naturally, the desired system parameters depend on the route of administration, the site of application, and several other factors. Modern pharmaceutical development based on continuous consideration of finished-product properties is carried out according to the Quality by Design (QbD) paradigm. However, applying this strategy, which has gained increasing industrial relevance over the past two decades, to complex stimulus-responsive systems such as PIISI requires detailed knowledge of the physicochemical properties of every matrix component and of their individual effect on the target product profile. At present, such information is insufficient to provide a unified methodological framework supporting the rational development of new PIISI-based drug products.

Among the candidate matrix-forming polymers, bleached shellac was selected for the present study based on a combination of properties that distinguish it from the more widely used PLGA. Shellac is a natural oligomeric resin (Mw ~1000–2000 Da) bearing –COOH, –OH and ester functional groups that govern both its solubility profile in polar aprotic and protic solvents and its insolubility in aqueous media at physiological pH [13,14,15]. It is biodegraded in vivo by endogenous esterases over a time scale (weeks to months) compatible with the 7–21-day release window required for periodontal and alveolar socket drug delivery, has GRAS regulatory status (FDA 21 CFR 175.300; included in the European, United States and Japanese Pharmacopoeias), and is industrially available at approximately 10–20-fold lower cost than pharmaceutical-grade PLGA. Its –COOH and –OH groups additionally enable mucoadhesive interaction with mucin glycoproteins, which is advantageous for retention in the periodontal pocket without the need for additional bioadhesive excipients [13]. PLGA, despite its excellent track record, suffers from acid-catalyzed degradation that lowers local pH and may aggravate periodontal inflammation; because of its high cost requirements, often medical-grade NMP solvent systems complicate clinical translation and scale-up. The physicochemical and regulatory rationale for shellac is summarized in detail in Appendix A.

In addition to matrix selection, the field still faces several unresolved challenges that frame the present work. First, residual organic solvents in PIISI vehicles (NMP, DMSO, BA, EL) can be cytotoxic and locally irritating, and their concentration and rate of extraction must therefore be tightly controlled. Second, an uncontrolled burst-release effect is observed in the majority of phase-inversion systems and remains the principal pharmacokinetic limitation of this technology. Third, despite more than two decades of clinical use of Atridox^®^, no systematic methodological framework currently links the physicochemical properties of the matrix former and solvent system to the critical quality attributes of the resulting depot, which limits rational formulation development.

We therefore hypothesized that, for a bleached shellac-based PIISI, the critical quality attributes of the depot (phase-inversion time, implant structure, solvent diffusion volume and swelling) are primarily governed by the donor/acceptor balance of hydrogen-bonding interactions between shellac functional groups and the solvent system, and that this balance can be tuned through the rational pairing of polar aprotic (H-bond acceptor) and polar protic (H-bond donor) solvents. The aim of this study was to systematically investigate, within a Quality by Design framework, the influence of solvents, solvent combinations, polymer concentration, and excipients on the critical quality attributes of bleached shellac-based phase-inversion in situ implants, and, on this basis, identify a lead PIISI composition as a promising preliminary physicochemical platform for future dental drug delivery applications, pending biological validation.

## 2. Materials and Methods

### 2.1. Study Objects

**Figure 1:**
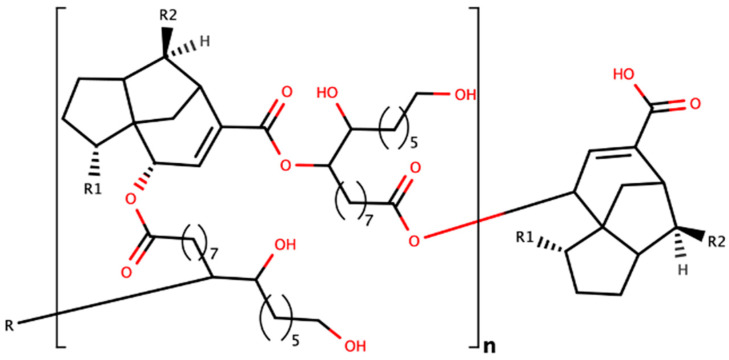
Structural formula of shellac.

Bleached shellac (Figure 1), a natural, biodegradable, and non-toxic oligomeric biopolymer approved for pharmaceutical use in most countries worldwide, was used as the matrix-forming polymer. In pharmaceutical practice, shellac is most commonly employed as a tablet film-coating component and as a matrix former for modified-release dosage forms [13,14,15]. It is industrially available and considerably less expensive than pharmaceutical-grade PLGA, which makes it an attractive candidate for scale-up of PIISI formulations.

To develop the optimal PIISI composition, the following solvents were selected and studied: N-methylpyrrolidone (NMP), dimethyl sulfoxide (DMSO), propylene glycol (PG), benzyl alcohol (BA), ethyl lactate (EL), triethyl citrate (TC), and benzyl benzoate (BB). In published studies, combinations of the aforementioned solvents are frequently used (Table 1). Regulatory status of each solvent is provided according to the following pharmacopoeial and regulatory references: U.S. Food and Drug Administration Inactive Ingredient Database (FDA IID); United States Pharmacopeia (USP); European Pharmacopoeia (EPh, also Ph. Eur.); Japanese Pharmacopoeia (JPh); and the Pharmacopoeia of the Eurasian Economic Union (PhEEU).

### 2.2. Quality by Design

According to the Quality by Design (QbD) approach, the initial stages of pharmaceutical development consist in defining the Quality Target Product Profile (QTPP) and identifying the corresponding Critical Quality Attributes (CQAs) against which the formulation must be evaluated.

QTPP in the form of PIISI, depending on the method of its application in dentistry, is presented in Table 2. These target parameters were determined according to the optimal compositions in published data and were based on the clinical experience of dentists and specialists’ requirements for the drug delivery system.

Alveolar socket and periodontal pocket implants differ chiefly in the requirements for structural and mechanical strength. An alveolar socket implant must hold a stable shape and exhibit defined ultimate strength and elastic modulus in order to prevent socket overgrowth and allow for subsequent dental implantation. A periodontal pocket implant, in contrast, requires a more elastic structure that does not cause mechanical injury to soft tissues. The set of CQAs that is common to both applications and was used in the present study is summarized in Table 3.

### 2.3. Study Design

**Figure 2:**
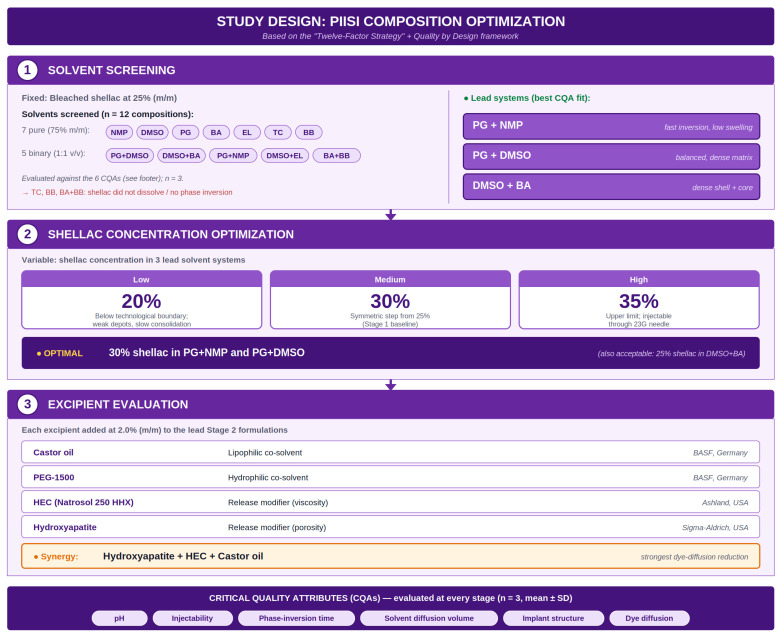
Study design: the three-stage screening strategy used in this work. Stage 1: Bleached shellac fixed at 25% (m/m); twelve combinations of seven neat solvents (NMP, DMSO, PG, BA, EL, TC, BB) and five 1:1 binary mixtures (PG+DMSO, DMSO+BA, PG+NMP, DMSO+EL, BA+BB) screened against the predefined CQA set (injectability, phase-inversion time, solvent diffusion volume, implant consistency and structure). Stage 2: Shellac concentration varied at three levels (20, 30 and 35% m/m) in the three lead solvent systems retained from Stage 1 (PG+NMP, PG+DMSO, DMSO+BA); 25% was not re-tested at this stage because it had already been characterized in Stage 1. Stage 3: Addition of release-modifying excipients (HEC, PEG-1500, hydroxyapatite, castor oil, and the HA + HEC + castor oil combination) at a constant 2% (m/m) to the lead Stage 2 formulations. All materials used in Figure 2 are listed in Section 2.1 and Section 2.3.

This study was organized in three sequential stages (Figure 2), following the previously proposed “Twelve-Factor Strategy” [28] for the systematic prevention of burst release in depot formulations produced by phase-inversion in situ technology.

According to that strategy, at least twelve groups of factors affect API release from PIISI matrices: five are related to the matrix former, two to the API itself, and the remainder to the overall composition (matrix-forming polymer concentration, polymer combinations, use of co-solvents, and addition of release-modifying excipients) [28].

In the first stage, the matrix former concentration was kept constant at 25.0% (m/m), a value previously identified as an average optimum for shellac-based PIISI [30], and solvents and binary solvent combinations were screened. Twelve compositions were prepared in 75.0% (m/m) of either a single solvent (NMP, DMSO, PG, BA, EL, TC or BB) or a 1:1 (*v*/*v*) binary mixture (PG+DMSO, DMSO+BA, PG+NMP, DMSO+EL or BA+BB). All twelve compositions were evaluated against the CQA set defined in Table 3, with target specifications taken from previously published experimental studies.

In the second stage, the influence of matrix-former concentration on the CQAs was investigated for the lead solvent combinations identified in Stage 1 (Table 2). Three concentration levels of bleached shellac were tested: low (20.0%), medium (30.0%) and high (35.0%) (m/m). The selection of these three concentration levels was guided by the following considerations: (i) 20% (m/m) represents the lower technological boundary, below which shellac concentrations have previously been shown to yield mechanically weak depots that fail to form a coherent implant within the agar pocket model in less than 4 h [30]; (ii) 30% (m/m) reflects the central level on either side of the 25% (m/m) baseline that was fixed in Stage 1, providing symmetric exploration of the concentration domain; and (iii) 35% (m/m) corresponds to the upper boundary at which the solvent fraction (≤65%) is still sufficient to dissolve shellac without precipitation upon cooling and to maintain an injectability force compatible with a 23G needle in preliminary trials. The 20–35% range is also consistent with the intervals reported for other natural-resin and PLGA-based PIISI formulations in the recent literature [11,30], and was deliberately kept narrow because, as a univariate screening rather than a full design of experiments, our purpose at this stage was to identify the inflection point at which CQA behaviour changes, rather than to construct a multivariate response surface (this limitation is explicitly addressed in Section 4). All samples were characterized with respect to the same CQA panel: dissolution behaviour, stability of the liquid PIISI vehicle, phase-inversion rate, injectability, mechanical strength of the formed implant and dye diffusion.

In the third stage, the effect of additional excipients on the CQAs was assessed. The excipients tested included a lipophilic co-solvent (castor oil, BASF SE, Ludwigshafen, Germany), a hydrophilic co-solvent (PEG 1500, BASF SE, Ludwigshafen, Germany), and two release-modifying agents: hydroxyethylcellulose (Natrosol 250 HHX, Ashland, OR, USA) and hydroxyapatite (Sigma-Aldrich, St. Louis, MO, USA). All samples were analyzed against the same predefined CQA panel.

The study design is schematically presented in Figure 2.

### 2.4. Preparation of Solutions

To prepare phase-sensitive PIISI matrices, bleached shellac was dissolved in a weighed amount of the chosen solvent (or solvent combination) at 70 °C. Dispersion was performed on an IKA^®^ C-MAG HS 7 (IKA-Werke GmbH & Co. KG, Staufen, Germany) digital magnetic stirrer at medium speed for 1 h. The resulting vehicle was transferred to sealed conical tubes and treated in a VBS-3DP ultrasonic bath (Vilitek LLC, Moscow, Russia) for 30 min. After cooling to room temperature (22–25 °C), excipients were added to the homogeneous vehicle, and stirring on the magnetic stirrer was continued at room temperature (22–25 °C) at medium speed until complete dissolution.

### 2.5. Methods for Critical Quality Attributes Determination

To assess the influence of the medium on the solubility of the matrix former, the apparent pH of each liquid PIISI vehicle was measured prior to phase transition using a Starter 2100 pH metre (OHAUS, Parsippany, NJ, USA).

Injectability was assessed using the method described in [10]. A syringe containing 1 mL of the test sample was fitted with a 23-gauge needle and mounted in a TA.XT*plus* texture analyzer (Stable Micro Systems, Godalming, UK). The upper probe was driven downwards at 1.0 mm/s with a trigger force of 0.1 N until it reached the base of the barrel at room temperature (22–25 °C). Injection work (N·mm) and peak injection force (N) were recorded over a 10 mm displacement in five independent replicates (n = 5).

To assess the swelling of the formed PIISI, samples measuring 1 cm^3^ were placed in 10 mL of phosphate buffer (pH 6.8) for 90 min, after which changes in sample volume were recorded. In most published studies investigating the swelling of shellac- and PLGA-based implants, this parameter was studied over a 24–48 h period [31]. However, expert opinion of clinical dentists demonstrates that the most critical volume changes of materials placed in the socket or pocket occur during the first hours after the procedure, justifying a shorter experimental time. In addition, the duration was selected to match the expected residence time of the liquid PIISI vehicle in the periodontal pocket prior to its full consolidation, since shellac-based implants reach a structurally stable state within 60–90 min (see Section 3.1) and any further volume changes occur within an already solidified matrix that no longer interacts with the periodontal tissues in a clinically relevant manner. The experiment was performed in five independent replicates (n = 5).

Phase inversion was studied using two complementary approaches: a standard in vitro precipitation test in phosphate buffer and an agar block model (Section 2.6). Following the established method described in [10], a 1 mL aliquot of the sample was loaded into a 3 mL plastic syringe and injected through an 18-gauge needle into 5 mL of phosphate buffer (pH 6.8) in a glass vial. Phase inversion was monitored visually and photographed at 5, 15, 30 and 60 min after injection. As in previous experiments, phase inversion also was studied in five independent replicates (n = 5).

A simplified in vitro periodontal pocket model based on cylindrical 6 mm cavities drilled in agar blocks has been previously described [32] and was used in the present work to study matrix formation.

### 2.6. Agar Model for In Vitro

The agar models were prepared from a 4.0% (*w*/*v*) agar solution in phosphate buffer (pH 6.8). Agar was gradually dissolved in the required volume of buffer under stirring at 80 °C, brought to the boil, and boiled for 5 min, and the resulting viscous, homogeneous liquid was poured into silicone moulds (3.3 × 3.3 × 2.0 cm).

For the alveolar socket models, casts of a two-rooted tooth were positioned strictly vertically inside the silicone moulds, which were then filled with agar solution to a volume of 22 cm^3^ (up to the mark). The blocks were left to cool and refrigerated until complete solidification, and the tooth casts were then removed from the wells. The internal cavity volume (400 ± 25 μL) was verified using an automatic pipette.

For the periodontal pocket models, slit-shaped openings of approximately 100 μL volume, mimicking the geometry of a periodontal pocket [33], were cut in the agar block with a laboratory spatula.

Before testing, the agar wells were sprayed with phosphate buffer and then filled with the experimental compositions, to which a water-soluble red dye had been added to enable visual assessment of the solvent diffusion front. Ten microlitres of phosphate buffer were applied to the surface of each sample to mimic the salivary environment, and the blocks were incubated at 37 ± 1 °C to reproduce oral-cavity temperature. The implants were periodically re-sprayed with buffer to maintain humidity. At predefined time points, the position of the dye diffusion front in the agar block and the degree of implant solidification were recorded. The dye-diffusion volume was estimated by 3D reconstruction in Tinkercad software (Autodesk Inc., San Rafael, CA, USA; web application, no version number; https://www.tinkercad.com, accessed on 15 December 2025): the measured front coordinates were entered into the software and used to construct geometrical primitives whose volume reflected the extent of solvent migration into the surrounding tissues [33].

### 2.7. Statistical Analysis

Each formulation was prepared and characterized in five independent replicates (n = 5) unless otherwise stated. All quantitative results are expressed as the arithmetic mean ± standard deviation (SD). Comparison of multiple formulations for a given critical quality attribute (injectability force, phase-inversion time, swelling degree, solvent diffusion volume) was performed by one-way analysis of variance (ANOVA) followed by Tukey’s post hoc test for pairwise comparisons. Differences were considered statistically significant at *p* < 0.05. Categorical/qualitative observations (e.g., implant integrity classification, presence or absence of phase inversion) were summarized as the modal observation across the five replicates and are reported descriptively. Statistical computations were performed in Microsoft Excel (Microsoft 365) using the Data Analysis Toolpak and verified in OriginPro 2021 (OriginLab Corp., Northampton, MA, USA).

## 3. Results

### 3.1. First Stage of the Study

In the first stage, a panel of seven neat solvents and five 1:1 (*v*/*v*) binary mixtures was evaluated at a constant shellac concentration of 25% (m/m).

Shellac was highly soluble in NMP, DMSO, and PG, as well as in the binary systems PG+DMSO, PG+NMP, and DMSO+BA, yielding homogeneous solutions. The dissolving capacity of BA, EL and the DMSO+EL mixture was lower. In TC and BB, shellac did not dissolve, and the polymer formed aggregates that could not be dispersed either by ultrasonic treatment or by heating.

NMP- and DMSO-based vehicles showed a gradual increase in viscosity over time. After heating, they could be passed through a 23G needle, although with noticeable resistance, as could the EL- and BA+BB-based vehicles. In contrast, the PG-based vehicle and its 1:1 mixture with DMSO and NMP retained stable fluidity, showed no tendency to thicken, and were freely drawn into a syringe through a 23G needle. The shellac solution in pure BA remained highly viscous and could not be expelled through a 23G needle, even after additional heating (Table 4).

A characteristic feature of certain solvent–polymer systems was a spontaneous increase in viscosity at rest. This effect was observed for the DMSO-, NMP-, and EL-based vehicles within 5 min after preparation, and an additional pre-injection heating step was therefore introduced into the manufacturing protocol. From the standpoint of solvent solubility power, PG was less effective than NMP and DMSO; however, the PG-based vehicle showed no tendency to thicken on standing. In the binary mixtures PG+NMP and PG+DMSO, PG suppressed the spontaneous viscosity increase, while NMP and DMSO ensured higher shellac solubility than PG alone, combining the advantages of the two solvent classes.

The next experimental stage consisted of identifying the composition with the best precipitation properties.

When the shellac solutions were injected into phosphate buffer, PIISI formation proceeded by solvent exchange, with the rate of phase inversion and the consistency of the resulting implant depending on the nature of the solvent (Table 5). The DMSO-based system formed an implant within approximately 1 min, with a dense outer shell and a uniformly dense core; similar behaviour was observed for the PG+NMP system. The NMP-based system also formed a dense outer shell within 1 min, but the core remained liquid; a comparable formation time with a liquid core and a soft shell was observed for the DMSO+EL system. The DMSO+BA system produced a dense, structurally homogeneous implant within 1–2 min. The pure PG system solidified more slowly (2–3 min) and yielded a solid but brittle implant. In the PG+DMSO system, an implant with a dense shell and a gel-like core formed within 1–2 min. The shellac solutions in pure BA and pure EL produced implants only after 5 min, with no well-defined outer shell and a soft shell with a gel-like core, respectively. In the BA+BB system, no phase inversion occurred; the composition remained liquid throughout the 30 min observation period.

Three solvent systems were selected for further evaluation of the in situ implants: PG+DMSO, PG+NMP and DMSO+BA. These three systems provided adequate shellac solubility, vehicle stability on storage, convenient injectability through a 23G needle, and reproducible formation of well-defined implants in phosphate buffer.

For these three lead systems, the rate of in situ implant formation was assessed in agar blocks simulating a periodontal pocket. The fastest and most distinct implant formation was observed for the PG+NMP and PG+DMSO systems: after 30 min, both produced hard, dense, and structurally homogeneous implants. In the DMSO+BA system, a structured implant had formed by 30 min, but its outer shell remained soft and elastic; complete solidification took up to 3 h.

**Figure 3:**
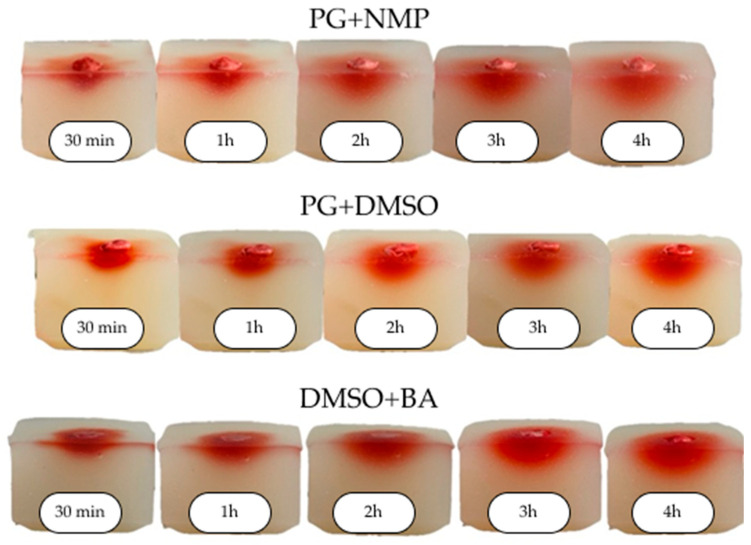
Dye diffusion from implants at 30 min, 1 h, 2 h, 3 h, and 4 h.

The diffusion of the dye from the implants into the agar periodontal pocket model was then evaluated. During the first hour, dye release was more pronounced for the DMSO+BA system than for PG+NMP or PG+DMSO; however, the diffusion rate of the DMSO+BA system subsequently decreased, presumably as a result of progressive matrix densification. After 4 h, the largest dye-diffusion zone was recorded for the PG+NMP system (Figure 3; Table 6).

The degree of implant swelling was also evaluated. Over 90 min, the highest swelling was observed for the PG+DMSO system, which absorbed 0.7 mL of buffer corresponding to an approximately 71% increase in volume. The DMSO+BA system showed moderate swelling of 42%, whereas the PG+NMP system showed the lowest absorption with only a 22% increase in volume (Table 6).

### 3.2. Second Stage of the Study

In the second stage, the effect of shellac concentration on the properties of the liquid vehicle and on the resulting PIISI was evaluated. Shellac solutions at 20%, 30% and 35% (m/m) were prepared in each of the three lead solvent systems and characterized against the same CQA panel.

For the DMSO+BA system, increasing the shellac concentration above 25% led to a sharp rise in viscosity: at 30% and 35%, a stable gel-like structure formed and persisted even after prolonged heating, precluding injection. Conversely, lowering the shellac concentration to 20% reduced viscosity, allowing for the vehicle to remain fluid, to be injected without pre-heating, and to pass freely through a 23G needle. In all systems, however, shellac concentrations of 20% and below produced structurally stable in situ implants only after 4 h. For the PG+NMP and PG+DMSO systems, increasing the shellac concentration to 30% (m/m) yielded the best balance of vehicle properties and in situ implant characteristics: complete solidification in the agar periodontal pocket model occurred within approximately 15 min. A further increase in shellac concentration to 35% (m/m) in the PG+NMP and PG+DMSO systems was associated with markedly higher viscosity and a prolonged implant-formation time of up to 50 min (Table 7).

### 3.3. Third Stage of the Study

For the final study stage investigating the effect of excipients on PIISI characteristics, three compositions were selected: a 25% shellac solution in DMSO+BA, as well as 30% shellac solutions in PG+NMP and PG+DMSO. Each sample was supplemented with hydroxyapatite (HA), hydroxyethylcellulose (HEC), PEG-1500, castor oil (BASF SE, Ludwigshafen, Germany; described in Section 2.3), and the HA + HEC + castor oil combination at a constant concentration of 2%.

The addition of HA to all formulations produced implants with a heterogeneous, porous structure and increased brittleness. In the PG+NMP and PG+DMSO systems, HA did not appreciably alter the implant-formation rate, whereas in the DMSO+BA system, the solidification time was reduced to 1 h. In all cases, the presence of HA increased the rate of dye diffusion.

The addition of HEC made the shellac solutions more viscous and slowed both implant formation and dye release. The effect was most pronounced in the DMSO+BA system, in which solid implant formation was not observed even after 2 h and the matrix remained gel-like. PEG-1500 produced an effect similar to that of HEC and additionally yielded softer, more plastic implants.

Introduction of castor oil had a similar effect on the PG+NMP system, increasing solid implant formation time to 1 h and reducing dye diffusion rate. In contrast, for PG+DMSO and DMSO+BA castor oil introduction did not significantly affect solidification rate and dye release from the implant.

**Figure 4:**
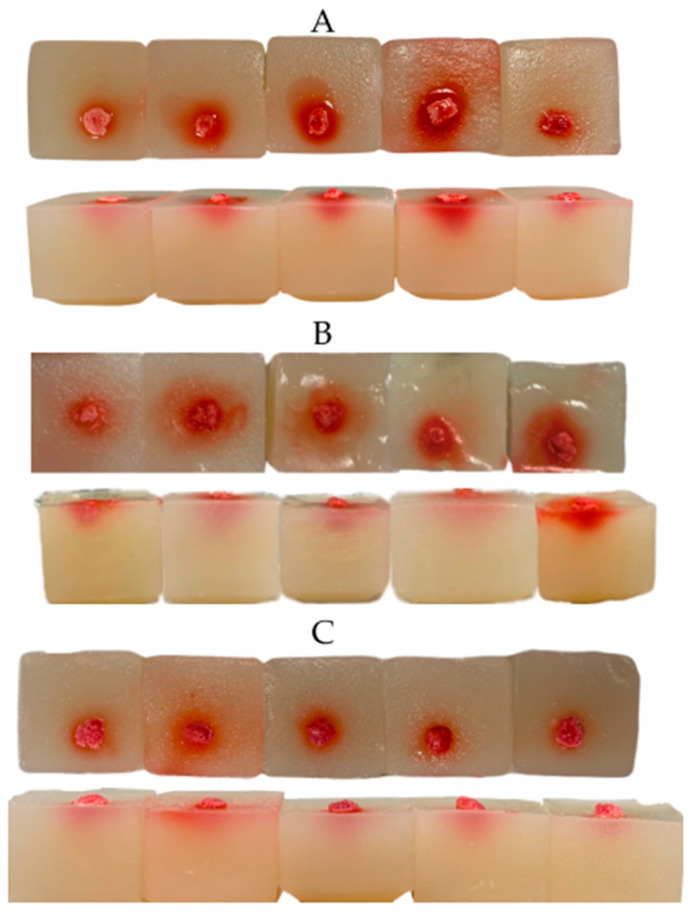
Effect of release-modifying excipients on the dye-diffusion behaviour of bleached shellac PIISI in the agar periodontal pocket model, recorded 2 h after injection. Each panel shows, from left to right, samples containing PEG-1500, hydroxyapatite (HA), hydroxyethylcellulose (HEC), castor oil and the combined HA + HEC + castor oil mixture (each at 2.0% (m/m)) in three solvent systems: (**A**) PG+DMSO, (**B**) PG+NMP and (**C**) DMSO+BA. Representative images of n = 5 replicates per condition.

The combined HA + HEC + castor oil mixture, added to all three lead systems, produced heterogeneous implants and the strongest reduction in dye-diffusion rate, indicating a synergistic effect of the three excipients (Figure 4).

## 4. Discussion

The present study examined how the principal compositional variables of a PIISI—the choice of solvent, the polymer concentration, and the additional excipients—influence the critical quality attributes of alveolar socket and periodontal pocket implants. Among candidate matrix formers for phase-inversion in situ implants, bleached shellac occupies a particular position. Unlike fully synthetic polymers such as PLGA, it is a natural oligomeric resin with more than a century of pharmaceutical use, yet its application in modern stimulus-responsive delivery systems has only recently received systematic attention. The rationale for selecting shellac as the matrix former of a dental PIISI therefore needs to address not only empirical performance but also the underlying physicochemical and biological properties that determine its suitability for the intended clinical use. A comprehensive overview of these characteristics, encompassing molecular composition, functional group chemistry, biodegradation kinetics, regulatory status, and cost, together with their explicit alignment to the QTPP defined in Section 2.2, is presented in Appendix A.

Our results are consistent with previously published studies on PLGA-based PIISI, which together confirm that the matrix former can be substituted without a major loss in technological performance, provided that the solvent system and the additional excipients (co-solvents, plasticizers, viscosity modifiers) are tuned to achieve the desired QTPP [28]. Vora et al. [29] introduced various hydrophilic polymers—hydroxypropyl methylcellulose (HPMC), hydroxypropyl cellulose (HPC), carbopol (Car) and carboxymethylcellulose (CMC)—into PIISI compositions based on NMP and PLGA, and studied their effect on implant formation and drug release. They reported that HPMC and Car effectively reduced burst release (the high API release seen during the first hours of testing), whereas HPC and CMC accelerated phase inversion and produced more porous implant morphology. In our hands, HEC behaves similarly to HPMC; the contrasting behaviour of other cellulose derivatives reported in [29] warrants further mechanistic investigation.

As discussed above, the requirements for implant consistency and the duration of API release differ between alveolar socket and periodontal pocket implants. On the basis of the present data, the more plastic compositions based on PG+DMSO or PG+NMP with PEG-1500 or HEC appear better suited to the periodontal pocket, whereas the denser structures formed by PG+NMP without modifying excipients are preferable for the alveolar socket. The actual API release kinetics from these matrices will depend on the physicochemical properties of the chosen drug substance and need to be assessed in dedicated release studies.

The differences in solubility, phase-inversion kinetics, and implant structure observed in Table 4 and Table 5 are interpreted in light of the intermolecular interactions expected between bleached shellac and each of the solvent systems employed. Below, the experimental results are systematically linked to a proposed mechanistic framework summarized in Table 8. It should be emphasized that this framework is consistent with the experimental observations and with the published Kamlet–Taft hydrogen-bond donor (α) and acceptor (β) parameters of the solvents involved, but constitutes a working hypothesis whose direct experimental validation by FTIR/Raman spectroscopy, DSC, and computational modelling is foreseen as the next step (see the Section Study Limitations and Future Work).

The behaviour of NMP and DMSO—two strong polar aprotic hydrogen-bond acceptors with comparable Kamlet–Taft parameters (β ≈ 0.76–0.77)—clearly demonstrates the dominant role of H-bonding in shellac solvation. Both solvents completely dissolved the polymer within 25–30 min and produced the highest pH values of the initial solutions (12.84 and 11.27, respectively), reflecting effective deprotonation of the carboxylic groups of shellac upon solvation. The carbonyl oxygen of NMP and the sulfoxide group of DMSO accept protons from the –OH and –COOH groups of the polymer, disrupting the intermolecular self-association network. However, it is precisely this strong solvation that caused both systems to exhibit a tendency toward spontaneous viscosity increase upon standing and required additional heating prior to injection. The difference in the structure of the resulting implants (Table 5)—a “dense shell with liquid core” for NMP versus a uniformly dense matrix for DMSO—is explained by the higher water miscibility of DMSO and, consequently, more uniform solvent extraction from the entire volume of the droplet, whereas NMP leaves the surface layer faster than the interior.

PG, as a polar protic solvent, acts on a fundamentally different principle: it solvates shellac through mutual hydrogen bonding without disrupting polymer chain entanglements as aggressively. This simultaneously explains experimental observations: the homogeneous solution does not thicken upon storage, passes freely through a 23G needle (≤10 N, Table 3), but forms an implant more slowly—150 ± 17 s compared with 60 s for NMP/DMSO—and the final structure is dense yet brittle (Table 5). The brittleness reflects the retention of a more ordered, crystallite-like chain organization after solvent removal.

BA and EL exhibit intermediate behaviour, limited by a weak donor (BA) or exclusively acceptor (EL) H-bonding profile. Both produced viscous solutions with dissolution times of 40 ± 10 min and formed implants within 280–290 s with a poorly defined structure (Table 4). For BA, an additional factor is its limited water miscibility, which slows the solvent-exchange process, whereas for EL, the absence of proton-donor groups prevents complete disruption of polymer self-association.

TC and BB—bulky esters with low acceptor strength and a pronounced lipophilic character—failed to dissolve shellac altogether (Table 4); the steric hindrance of TC and the van der Waals nature of BB interactions render mixing thermodynamically unfavourable. Accordingly, these systems were excluded from further consideration.

The binary combinations proved to be the most informative. The PG+NMP and PG+DMSO (1:1) systems realize the synergy between a donor (PG) and an acceptor (NMP/DMSO): the aprotic component ensures dissolution, while PG suppresses uncontrolled viscosity growth, manifested in stable homogeneous solutions with pH 8.4–9.95 and free injectability without preheating (Table 4). Both combinations deliver the fastest and most reproducible phase-inversion results: 50 ± 3 s for PG+NMP and 90 ± 10 s for PG+DMSO, with the PG+NMP system producing a structurally uniform, dense implant (Table 4), which corresponds to an optimal balance between the extraction rates of both components. The minimum swelling degree of this system (22%) provides additional confirmation that the resulting matrix possesses the most ordered structure with the fewest “defective” hydratable regions.

DMSO+BA (1:1) displays interesting intermediate behaviour: BA, acting as a moderate H-bond donor, slows the extraction of DMSO by water, which leads to the formation of a more uniform implant within 90 ± 10 s without a pronounced “skin effect”. This explains why this combination was selected among the leading three, despite the initial solution viscosity.

Finally, the two “failed” combinations illustrate the importance of the donor/acceptor balance. DMSO+EL contains two acceptors without a donor; the mechanistic profile is redundant and of a single type, resulting in a soft shell and liquid core (Table 4) and yielding a practically unusable structure. BA+BB combines two low-polarity aromatic solvents, which fails to ensure homogeneous dissolution of shellac, rendering phase inversion thermodynamically impossible; the composition remained liquid throughout the full 30 min of observation.

**Figure 5:**
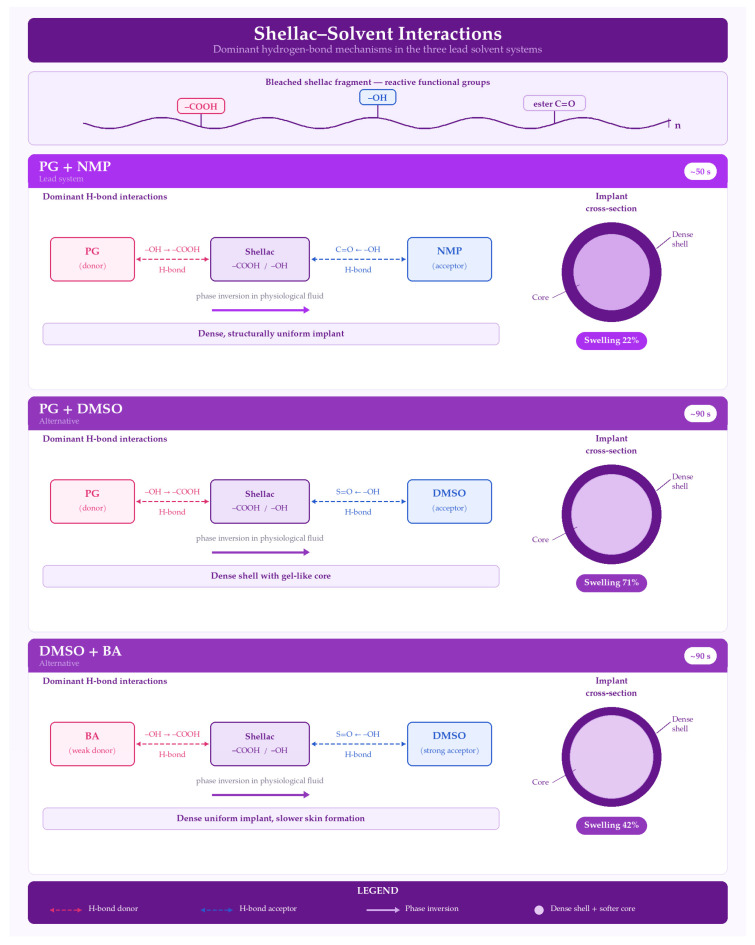
Schematic representation of the proposed hydrogen-bond interactions between bleached shellac (–COOH, –OH and ester C=O groups) and the three lead solvent systems (PG+NMP, PG+DMSO and DMSO+BA), and their relationship with the experimentally observed phase-inversion times, implant cross-section, and swelling degree. The diagram is illustrative and is intended as a working hypothesis; direct spectroscopic and computational validation is foreseen as the next step (see the Section Study Limitations and Future Work).

To visually summarize the mechanistic framework outlined in Table 8, Figure 5 schematically depicts the dominant hydrogen-bond interactions between the reactive functional groups of bleached shellac (–COOH, –OH, ester C=O) and the three lead solvent systems (PG+NMP, PG+DMSO, DMSO+BA). Each panel links the donor/acceptor pairing to the observed phase-inversion time, the resulting implant cross-section, and the experimentally measured swelling degree.

Thus, the entire experimental dataset can be rationalized through a single unifying principle: an optimal PIISI composition requires the combination of a strong H-bond donor and a strong H-bond acceptor with sufficient water miscibility; all three conditions are simultaneously satisfied in the PG+NMP system, which explains its selection as the lead formulation.

**Figure 6:**
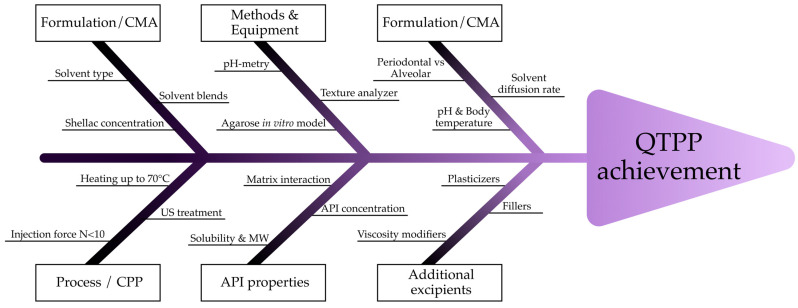
Ishikawa diagram.

At this stage, based on the obtained data, after studying a wide range of solvents theoretically suitable for PIISI, as well as excipients of varying hydrophilicity and functionality, their contributions to achieving the quality target product profile can be assessed (Table 9) and the main development focus areas can be formulated (Figure 6).

### Study Limitations and Future Work

Several limitations of the present study should be acknowledged explicitly so as to delimit the scope of the conclusions and define the priorities for subsequent investigations. (i) Scope of the QbD framework: The present work implements the QTPP and CQA stages of the Quality by Design paradigm and uses a structured univariate screening of solvents, polymer concentrations, and excipients across the three sequential stages defined in Figure 2. It does not, however, include a formal multivariate Design of Experiments (DoE), response-surface modelling, or quantitative risk assessment with criticality scoring; the Ishikawa diagram (Figure 6) and the CMA/CQA matrix (Table 9) are therefore presented as illustrative QbD tools that map the design space rather than as the output of a full QbD optimization. A subsequent study, which is in preparation, will apply a D-optimal mixture design to the three lead solvent systems with shellac concentration and selected excipients (HEC, PEG-1500, castor oil, hydroxyapatite) as continuous and categorical factors. (ii) Mechanistic interpretation: The hydrogen-bond donor/acceptor framework summarized in Table 8 is consistent with all observed CQA trends and with the published Kamlet–Taft solvatochromic parameters of the studied solvents, but it is, at this stage, a working hypothesis based on indirect evidence. Direct confirmation will require complementary techniques—in particular FTIR and Raman spectroscopy of liquid and solidified shellac–solvent systems to localize H-bond donor and acceptor shifts of the –OH, –COOH, and ester C=O bands, differential scanning calorimetry (DSC) and modulated DSC of the dry implants to verify the changes in glass transition behaviour, and, ideally, molecular dynamics or DFT modelling of representative shellac model compounds (e.g., aleuritic acid) with NMP, DMSO, and PG. The corresponding work is currently being planned. (iii) In vitro models and surrogate markers: The agar periodontal pocket and alveolar socket models employed here, although consistent with the prior literature [32,33], are simplified physical surrogates that do not reproduce the cellular and proteinaceous environment of the periodontium. Phase inversion was assessed by visual inspection and 3D reconstruction of the dye diffusion front, both of which retain a degree of subjectivity; image-analysis quantification of the diffusion front (e.g., densitometric profiling, machine-vision based contour extraction) is being considered for future work. Likewise, the use of a water-soluble dye as a surrogate for an active pharmaceutical ingredient (API) provides information about solvent extraction kinetics and matrix porosity, but cannot directly substitute for an API release profile, since the partition behaviour and molecular volume of the surrogate differ from those of typical periodontal APIs (doxycycline, minocycline, chlorhexidine, meloxicam). Validated in vitro release studies with these APIs in compendial dissolution apparatus and in flow-through cells are planned as the next step. (iv) Long-term stability and mechanical performance: The present work has characterized the early phase-inversion phenomena (up to 4 h) and the short-term swelling (90 min) of the depots, in line with the clinically relevant time window during which the implant is most vulnerable to displacement and most prone to burst release. Long-term stability of the liquid vehicle (ICH-compliant accelerated stability at 25 °C/60% RH and 40 °C/75% RH for 6–12 months) and prolonged release studies (up to 21 days, with calculation of cumulative release, fitting to Higuchi/Korsmeyer–Peppas/zero-order kinetics, and assessment of mass loss/erosion of the matrix) will be reported in the subsequent communication. The mechanical properties of the formed implant (Young’s modulus, ultimate compression strength, elastic recovery) will be measured by texture analysis and dynamic mechanical analysis on the same set of formulations. (v) Biocompatibility: All matrix and solvent components used in the present formulations have established regulatory status (Table 1; Section 1), and shellac has GRAS designation (FDA 21 CFR 175.300) with a long history of safe oral use. NMP, DMSO, PG and BA are widely used parenteral solvents within the limits set by the FDA Inactive Ingredient Database. However, the present study did not include direct biological evaluation of the formulations: in vitro cytocompatibility on human gingival fibroblasts and periodontal ligament cells (MTT, LDH, live/dead staining), pro-inflammatory cytokine response (IL-1β, IL-6, TNF-α) on macrophage cell lines, and ex vivo testing on porcine periodontal tissue are foreseen for the next stage of development, and are required prior to in vivo evaluation. (vi) Comparison with commercial systems: A direct head-to-head comparison with Atridox^®^ (PLGA/NMP, 36.7%/63.3%) under identical experimental conditions was not performed in this study, but is planned as a benchmarking experiment in the subsequent work, alongside formulations based on borneol and sucrose acetate isobutyrate—the principal alternative natural-origin matrix former reported in the recent literature for periodontal PIISI [5,8,12].

## 5. Conclusions

PIISI continue to attract considerable scientific interest as effective stimulus-responsive drug delivery systems offering high stability and good prospects for scale-up and commercialization. One of the few approved drug products based on in situ technology—the Atrigel^®^ platform—is a PIISI and has been in clinical use for more than two decades. Despite this success, the translation of new in situ-based formulations into clinical practice has remained limited.

The PIISI literature remains fragmented: most reports describe isolated successful formulations rather than systematic mechanistic studies. As a consequence, it is difficult to draw make robust general conclusions about the effectiveness of the proposed solutions or about the transferability of methods and compositions to formulations containing other APIs. While the role of solvents and matrix formers in PIISI is well recognized in principle, their combined effect on the critical quality attributes of the resulting depot has not yet been comprehensively assessed.

In the present work we have undertaken a structured assessment of how the choice of solvent system and the addition of hydrophilic and hydrophobic excipients influence the principal parameters of phase-inversion stimulus-responsive systems: pH of the liquid vehicle, injectability, phase-inversion time, consistency and strength of the forming in situ implant, and solvent diffusion volume.

The best overall balance of shellac solubility, vehicle stability, ease of injection through a 23G needle, rapid implant formation and controlled dye diffusion was achieved with a 30% (m/m) shellac solution in PG+NMP. The excipient screening further showed that HEC and PEG-1500 at 2.0% (m/m) modulate the dye-diffusion rate without compromising implant formation kinetics or structural integrity.

Future work will focus on the dose-dependence of the excipient effects on the CQAs and on the use of advanced imaging methods (e.g., micro-computed tomography and confocal microscopy) for detailed visualization of the internal architecture of the forming implant.

In quantitative comparison with previously reported PIISI systems, the lead 30% shellac/PG+NMP formulation developed in this work delivers a phase-inversion time of approximately 50 s, which is faster than the corresponding values reported for cholesterol/NMP (90–180 s) [32] and borneol/NMP (60–120 s) [5] systems and comparable to the early stages of phase inversion of the PLGA/NMP-based Atrigel^®^ platform that underpins the clinically established Atridox^®^ product [11]. The 22% swelling of the lead formulation at 90 min is markedly lower than the values typically reported for shellac- and PLGA-based PIISI in the same buffer system (40–70%) [13,15,31], which is favourable for retention in the periodontal pocket. Critically, the use of bleached shellac instead of PLGA simultaneously addresses the high cost (~10–20-fold reduction per kilogram of pharmaceutical-grade polymer) and the local acidification associated with PLGA degradation, while preserving comparable phase-inversion performance and demonstrating compatibility with both hydrophilic (HEC, PEG-1500) and hydrophobic (castor oil, hydroxyapatite) modifiers. The present study establishes the 30% shellac/PG+NMP formulation as a promising preliminary physicochemical platform for dental drug delivery, with satisfactory in vitro technological performance across all evaluated CQAs. However, transition to a validated formulation for direct clinical application requires biological evaluation that was outside the scope of the present work; the immediate next steps, as outlined in the Section Study Limitations and Future Work, are therefore: (i) in vitro cytocompatibility studies on human gingival fibroblasts and periodontal ligament cells; (ii) assessment of pro-inflammatory cytokine response; (iii) FTIR/DSC validation of the proposed H-bonding mechanism; and (iv) the application of multivariate Design of Experiments and in vitro API release studies on the lead composition prior to in vivo evaluation.

## Figures and Tables

**Table 1:** Characteristics of solvents.

Solvent	Structural Formula	Regulatory Documentation
Dimethyl sulfoxide (DMSO) (Gaylord Chemical, USA, LA, Baton Rouge)	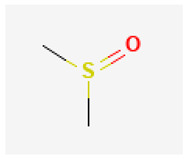	FDA, EPh, JPh, PhEEU [16]
N-methylpyrrolidone (NMP) (BASF, Germany, Ludwigshafen)	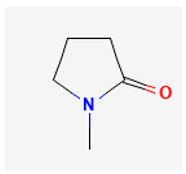	FDA, USP, EPh, JPh, PhEEU [16,17]
Propylene glycol (PG) (Dow Chemical, USA, TX, Freeport)	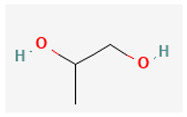	FDA, USP, EPh, JPh, PhEEU [17]
Benzyl alcohol (BA) (LANXESS, Germany, Leverkusen)	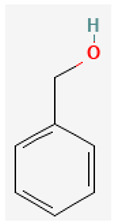	FDA, EPh, JPh [16,18]
Triethyl citrate (TC) (Vertellus, USA, IN, Indianapolis)	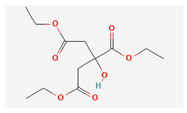	FDA, USP, EPh, JPh [17,19]
Benzyl benzoate (BB) (Emerald Kalama Chemical, USA, WA, Kalama)	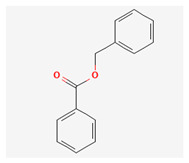	EPh, JPh [16]
Ethyl lactate (EL) (Corbion, Netherlands, Gorinchem)	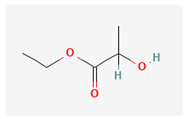	FDA [19]

**Table 2:** Quality Target Product Profile.

Characteristic	Alveolar Socket Implant	Periodontal Pocket Implant
Formation mechanism	Solvent diffusion (phase inversion)	
Phase transition time	Typically, after introduction into the socket, it is covered with a protective polymer membrane to prevent foreign particle entry. Final formation of PIISI under such conditions may take up to 2–3 h [20,21].	Since the periodontal pocket is often not surgically closed after drug administration, the formation of a dense structure resistant to evacuation from the pocket should occur in less than 0.5–2 h [22].
Injectability	Smooth injectability from a syringe of appropriate diameter, ensuring convenient administration [10,23].	
Biocompatibility	Absence of toxicity and inflammatory reactions to implant administration [24].	
Implant consistency and structure	Resistance to mechanical loads, external pressure, and elastic deformation [20].	Atraumatic properties, elasticity.
Mucoadhesion	Not applicable.	For an implant with a gel-like elastic consistency, mucoadhesion will be an important characteristic serving as an additional guarantee, beyond the structure and viscosity, of the drug remaining in the periodontal pocket for the designated time.
Swelling	Limited swelling of the formed in situ implant: up to 50% of the initial volume.	Limited swelling of the formed in situ implant: up to 50% of the initial volume.
Release API	Sustained release without burst effect over 7–21 days [25,26,27].	Sustained release without burst effect over 3–14 days [25,26,27].

**Table 3:** Critical Quality Attributes of the product.

Critical Quality Attribute	Target Specification	Justification/Rationale
Injectability	≤10.0 N	The optimal pressure range on the syringe plunger during injection was justified in study [5].
Phase inversion time	t_sedimentation_ ≤ 90 s t_consolidation_ ≤ 3 h	Formation of PIISI occurs gradually over several hours, with gradual solvent release from the matrix and its solidification.
Solvent diffusion volume	Retarded solvent diffusion over 4 h of observation in the agarose model of the alveolar socket	For PIISI a burst release type is often characteristic. To minimize side effects and prolong the effect, various excipients are introduced: viscous co-solvents, hydrophilic and hydrophobic polymers [28,29].
Implant consistency and structure	Formation of a dense implant shell with a solid porous or gel-like core	PIISI should be removable from the application site when necessary; its structure should ensure the planned modified release. A gel-like core and a dense formed shell will be advantageous for application in periodontal pockets; the system will be less traumatic, and the release will be less prolonged compared to solid implants.

**Table 4:** Study of CQA of shellac solutions depending on the solvent.

Solvent	pH	Dissolution Time of 25% (m/m) Shellac, min	Characteristics of the Obtained Solution	Injectability
DMSO	12.84	30 ± 7 a	Viscosity increases over time	After additional heating
NMP	11.27	25 ± 5 a	Viscosity increases over time	After additional heating
PG	8.10	35 ± 8 b	Homogeneous	≤10.0 N
BA	6.00	40 ± 10 c	Viscous	Does not pass
TC	5.96	Does not dissolve	**-**	**-**
BB	5.87	Does not dissolve	**-**	**-**
EL	4.73	40 ± 10 c	Viscous	After additional heating
PG+DMSO	9.95	30 ± 7 a	Homogeneous	≤10.0 N
DMSO+BA	8.47	30 ± 7 a	Viscosity increases over time	After additional heating
PG+NMP	8.40	35 ± 8 b	Homogeneous	≤10.0 N
BA+BB	4.73	40 ± 10 c	Viscous	After additional heating
DMSO+EL	6.59	40 ± 10 c	Viscosity increases over time	After additional heating

a—fast dissolution, b—moderate dissolution, c—slow dissolution (*p* < 0.05, Tukey’s post hoc test).

**Table 5:** Study of CQA of implants depending on the solvent.

Solvent	Implant Formation Time, s	Implant Characteristics
DMSO	60 ± 5 a	Dense structure throughout the volume
NMP	60 ± 5 a	Dense shell, liquid core
PG	150 ± 17 c	Dense, solid structure, but brittle
BA	280 ± 25 d	No distinct outer shell
EL	290 ± 25 d	Soft shell, gel-like core
PG+DMSO	90 ± 10 b	Dense shell, gel-like core
DMSO+BA	90 ± 10 b	Dense structure throughout the volume
PG+NMP	50 ± 3 a	Dense structure throughout the volume
BA+BB	Did not form	**-**
DMSO+EL	60 ± 5 a	Soft shell, liquid core

a—immediate formation, b—rapid formation, c—moderate formation, d—slow formation (*p* < 0.05, Tukey’s post hoc test).

**Table 6:** Swelling and solvent diffusion volume results for PIISI based on optimal solvent combinations.

Solvent	Solvent Diffusion Volume over 4 h, μL	Swelling, 90 min, %
PG+DMSO	385 ± 23 a	71.0 ± 7 a
PG+NMP	260 ± 15 b	22.0 ± 3 b
DMSO+BA	197 ± 9 c	42.0 ± 5 c

a—high level, b—low level, c—intermediate level (*p* < 0.05, Tukey’s post hoc test).

**Table 7:** Effect of shellac concentration on CQA PIISI based on optimal solvent combinations.

Solvent	Concentration	Solution Characteristics	Injectability, N	Implant Formation in Agar Model (min)
PG+NMP	20%	Homogeneous, fluid solution	≤10.0	240
	30%	Homogeneous, fluid solution	≤10.0	15
	35%	Viscous	23.0	50
PG+DMSO	20%	Homogeneous, fluid solution, passes through 23G needle	≤10.0	240
	30%	Homogeneous, fluid solution	≤10.0	15
	35%	Viscous	21.0	50
DMSO+BA	20%	Homogeneous, fluid solution	≤10.0	240
	30%	Stable gel-like structure	**-**	**-**
	35%	Stable gel-like structure	**-**	**-**

**Table 8:** Physicochemical interactions between bleached shellac and the studied solvents.

Solvent	Polarity/Class	Dominant Interaction with Shellac	Observed Effect on the System	Mechanistic Interpretation
NMP	Polar aprotic, strong H-bond acceptor (β ≈ 0.77)	Carbonyl O of NMP accepts H-bonds from shellac –OH and –COOH groups; dipole–dipole stabilization of acid groups.	Rapid, complete dissolution; viscosity increases over time; dense outer shell with liquid core after phase inversion.	Strong solvation breaks intermolecular H-bond network of shellac. Rapid water ingress upon injection causes immediate skin formation, while solvent retention inside delays full solidification.
DMSO	Polar aprotic, very high dipole moment (3.96 D), strong H-bond acceptor (β ≈ 0.76)	S=O of DMSO forms strong H-bonds with shellac –OH/–COOH; effective disruption of polymer self-association.	High solubility; viscosity rises with time; very fast phase inversion (~60 s) with uniformly dense implant.	Excellent thermodynamic solvent and high water miscibility cause both rapid initial dissolution and rapid solvent extraction by the surrounding aqueous medium, producing a uniformly precipitated matrix.
PG	Polar protic, hydroxylic; moderate dielectric constant (ε ≈ 32)	Mutual H-bonding via –OH groups; mild solvation of carboxyl groups.	Homogeneous fluid solution that does not thicken on standing; slower phase inversion (~150 s); brittle, dense implant.	PG behaves as a co-soluble protic solvent: it solvates shellac without disrupting polymer entanglements as aggressively as DMSO/NMP, leaving a more crystalline-like, brittle matrix after solvent loss.
BA	Aromatic alcohol, polar protic; moderate H-bond donor	Weak H-bonding via –OH; aromatic π-stacking with shellac terpenoid moieties.	Viscous solution; does not pass through 23G needle; slow implant formation (~5 min) with no defined shell.	Limited solvating power and limited water miscibility lead to slow solvent exchange, producing a soft, structurally undefined matrix unsuitable as a sole solvent.
EL	Polar aprotic ester; moderate polarity	Ester C=O accepts weak H-bonds from shellac –OH; no donor capability.	Partial dissolution; viscous solution; slow implant formation; soft shell with gel-like core.	Limited H-bond donor/acceptor balance gives only moderate solvation; weak entanglement disruption results in incomplete phase inversion.
TC	Polar aprotic ester; high MW, moderate polarity	Steric hindrance and low H-bond acceptor strength prevent effective shellac solvation.	Shellac does not dissolve; aggregate formation, not dispersible by ultrasound or heating.	Bulky triester is unable to penetrate the H-bonded shellac network; insufficient solvent–polymer interaction energy to overcome cohesive forces.
BB	Aromatic ester; lipophilic, very low polarity	Predominantly van der Waals/π-interactions; no H-bond donor capacity.	Shellac does not dissolve; persistent aggregates.	Lipophilic, non-polar character is incompatible with the highly polar shellac surface chemistry; thermodynamically unfavourable mixing.
PG+DMSO (1:1)	Combined polar protic + polar aprotic	Synergistic H-bond donor (PG) and acceptor (DMSO) interactions; PG suppresses excessive viscosification by DMSO.	Homogeneous, stable fluid solution; rapid phase inversion (~90 s); dense shell with gel-like core.	Balanced solvation: DMSO ensures dissolution while PG retards uncontrolled viscosity buildup. Differential extraction rates produce a structured shell–core implant ideal for periodontal pocket use.
PG+NMP (1:1)	Combined polar protic + polar aprotic	Same dual donor/acceptor mechanism as PG+DMSO; NMP provides slightly weaker but still strong solvation.	Stable homogeneous solution; very rapid phase inversion (~50 s); dense, structurally uniform implant; lowest swelling (22%).	Optimal solvent balance—high solubility, controlled solvent extraction, and minimal water uptake—explains the selection of this system as the lead formulation.
DMSO+BA (1:1)	Polar aprotic + weakly polar protic	DMSO dominates dissolution; BA contributes moderate H-bond donation.	Homogeneous solution after heating; phase inversion in 1–2 min; dense uniform implant; moderate swelling (42%).	BA modulates DMSO extraction kinetics, slowing skin formation and producing a more uniformly precipitated matrix; suitable as an alternative composition.
DMSO+EL (1:1)	Two polar aprotic	Both solvents are H-bond acceptors only; redundant interaction profile.	Viscosity rises over time; soft shell, liquid core; weak structural integrity.	Lack of H-bond donors limits the disruption of shellac self-association, resulting in incomplete network rearrangement after solvent removal.
BA+BB (1:1)	Aromatic protic + aromatic ester; both lipophilic	Insufficient polar solvation; no effective H-bond network with shellac.	No implant formation; composition remains liquid up to 30 min.	Combined low-polarity environment fails to dissolve shellac homogeneously; phase inversion is thermodynamically unfavourable.

**Table 9:** Effect of composition factors (CMA) on the main CQA PIISI.

	CQA	Injectability	Phase Inversion Time	Diffusion Volume	Implant Consistency and Structure
CMA					
PG		High	High	High	High
BA		Low	High	-	High
EL		Medium	High	High	High
HA		Low	Medium	High	Medium
HEC		High	High	High	Medium
PEG-1500		High	High	High	Medium
Oil		Low	Medium	High	Low

## Data Availability

The original contributions presented in this study are included in the article. Further inquiries can be directed to the corresponding author.

## Appendix A

**Table A1:** Physicochemical rationale for bleached shellac as a PIISI matrix former.

Property/Characteristic	Reported Value or Feature for Bleached Shellac	Relevance to PIISI for Dental Application
Origin and chemical nature	Natural resinous secretion of Kerria lacca; complex mixture of hydroxy fatty acids and sesquiterpenoid acids (aleuritic, jalaric, shellolic acids) linked by ester and lactide bonds.	Renewable, plant-/insect-derived biopolymer; aligns with sustainable polymer formulation trends and FDA/EU food/pharma approval.
Molecular weight	~1000–2000 Da (oligomeric); average degree of esterification ~70–80%.	Low molecular weight enables high solubility in polar aprotic solvents and rapid solvent exchange, supporting fast phase inversion (<2 h)—a key periodontal pocket QTPP requirement.
Functional groups	–COOH (acid value 65–75 mg KOH/g), –OH (hydroxyl value 230–260 mg KOH/g), ester C=O linkages.	Carboxyl and hydroxyl groups govern solubility behaviour, pH dependence, and hydrogen bonding with polar solvents (NMP, DMSO, PG); the same groups underlie pH-triggered precipitation in physiological fluids.
Solubility profile	Soluble in alcohols and polar aprotic solvents (NMP, DMSO, PG); insoluble in water, hydrocarbons, esters of high MW, and most aqueous buffers at pH < 7.	Ideal binary solubility profile for solvent-induced phase inversion: soluble in injectable vehicle, precipitates upon contact with aqueous tissue fluids.
Glass transition temperature (Tg)	≈40–45 °C (dry); decreases in presence of plasticizers.	Below body temperature (37 °C), the polymer is in a flexible state, enabling formation of an elastic implant suitable for the periodontal pocket without mechanical tissue trauma.
Film-forming and matrix-forming ability	Forms continuous, cohesive films and coherent solid matrices upon solvent removal.	Ensures formation of a stable depot capable of sustained-release drug delivery.
Biodegradability	Slowly hydrolyzed in vivo by endogenous esterases (weeks to months); degradation products are biocompatible fatty acids.	Matches the desired 7–21-day release window for alveolar socket implants and avoids the need for surgical removal.
Biocompatibility/safety	GRAS status (FDA 21 CFR 175.300); included in EPh, USP, JPh; established safety profile in oral and gastrointestinal use.	Regulatory pre-acceptance dramatically simplifies translation toward an approved dental drug product compared with newer synthetic biopolymers.
Cost and availability	Industrial-scale availability; ~10–20× cheaper than PLGA per kg of pharmaceutical grade.	Economically attractive for scale-up, addressing one of the main barriers to commercial PIISI translation.
Mucoadhesive potential	–COOH groups support hydrogen bonding and weak electrostatic interaction with mucin glycoproteins.	Supports residence in the periodontal pocket without the need for additional bioadhesive excipients.
Compatibility with co-excipients	Compatible with hydrophilic (PEG, HEC, HPMC) and hydrophobic (oils, fatty acids) modifiers.	Enables rational tuning of release rate and implant consistency, as demonstrated in the present study.
